# Virucidal Approaches for Hemorrhagic Fever Viruses

**DOI:** 10.3390/v17050663

**Published:** 2025-04-30

**Authors:** Raymond W. Nims, M. Khalid Ijaz

**Affiliations:** 1 Syner-G BioPharma, Boulder, CO 80503, USA; ray.nims@synergbiopharma.com; 2 Global Research and Development for Lysol and Dettol, Reckitt Benckiser LLC, Montvale, NJ 07645, USA

**Keywords:** arenaviruses, filoviruses, flaviviruses, hand hygiene, hantaviruses, nairoviruses, paramyxoviruses, phenuiviruses, surface inactivation, suspension inactivation

## Abstract

We have reviewed the primary literature on the virucidal efficacy of microbicidal active ingredients, formulated microbicides, and physical inactivation approaches (heat, irradiation) for hemorrhagic fever viruses (HFVs) (arenaviruses, filoviruses, flaviviruses, hantaviruses, nairoviruses, and phenuiviruses), and for two non-typical HFV paramyxoviruses. As each of these HFVs are large, lipid-enveloped RNA viruses, their susceptibilities to virucidal agents are informed by the so-called hierarchy of susceptibility of pathogens to microbicides. The unique susceptibility of lipid-enveloped viruses to most classes of microbicides is based on the common mechanisms of action of envelope-disrupting microbicides. Despite this, due to the relatively great lethality of these viruses, it is prudent (where possible) to confirm the expected efficacies of inactivation approaches in testing involving the HFVs themselves (as opposed to less lethal surrogate viruses) using field-relevant methods. Empirical data for virucidal activities of microbicidal active ingredients, formulated microbicides, and physical inactivation approaches, such as heat, ultraviolet light, and gamma irradiation, that were collected specifically for HFVs have been reviewed and summarized in this paper. These empirical data for surface and hand hygiene approaches, liquid inactivation approaches, and approaches for rendering diagnostic samples safe to handle inform non-pharmaceutical interventions intended to mitigate transmission risk associated with these HFVs.

## 1. Introduction

The hemorrhagic fever viruses (HFVs) are members of a group of virus families, including the *Arenaviridae*, *Filoviridae*, *Flaviviridae*, *Hantaviridae*, *Nairoviridae*, and *Phenuiviridae.* These, and two non-typical HFVs from the *Paramyxoviridae* family share the following commonalities [1]: they are relatively large, enveloped viruses with positive- or negative-sense single-stranded RNA genomes; they are arboviruses (insect-borne) or are carried in rodent or mammalian vectors; and they are uniquely lethal in terms of case mortality rates in humans. The U.S. Centers for Disease Prevention and Control (CDC) describes viral hemorrhagic fever as “a condition where many of the body’s organ systems are affected, the overall cardiovascular system is damaged, and the body’s ability to function on its own is reduced. In addition to VHFs, there are serious infectious diseases like Nipah and Hendra diseases that also require a specialized laboratory, are highly pathogenic, and have no, or limited, vaccine or treatment currently available. VHFs, Nipah, and Hendra disease can cause relatively mild illness or more life-threatening disease. Symptoms can vary but may include bleeding or hemorrhaging” [2]. Examples of specific HFVs include the arenaviruses Lassa virus, Junin virus, Machupo virus, Sabiá virus, Lujo virus, and Guanarito virus; the filoviruses Ebola virus and Marburg virus; the flaviviruses Kyasanur Forest disease virus, Alkhurma hemorrhagic fever virus, Omsk hemorrhagic fever virus, dengue virus, and yellow fever virus; the nairovirus Crimean–Congo hemorrhagic fever; the hantavirus Hantaan virus; and the phenuiviruses Rift Valley fever virus, heartland virus, and severe fever with thrombocytopenia syndrome virus [2,3,4]. The non-typical HFVs include the Nipah virus and Hendra virus [2]. Certain of the diseases associated with the HFVs to be discussed (including Crimean–Congo hemorrhagic fever, Ebola virus disease and Marburg virus disease, Lassa fever, Nipah disease and henipaviral disease, and Rift Valley fever) have been included in past World Health Organization (WHO) Priority Disease lists [5]. This inclusion signifies that these “diseases pose the greatest public health risk due to their epidemic potential and/or whether there is no or insufficient countermeasures” [5].

Due to the relative lethality of HFVs, manipulation of cell cultures infected with these viruses and handling of virus stocks for efficacy studies on inactivation approaches are typically restricted to BSL-4 laboratories. This has resulted in a slower accumulation of suspension- and surface-inactivation efficacy data for microbicides and efficacy data for hand/skin hygiene products for these viruses relative to viruses which are able to be handled safely in BSL-2 or BSL-3 laboratories. Fortunately, in the absence of such empirical data, one may predict the efficacy of microbicides for use against HFVs on the basis of the so-called hierarchy of susceptibility of pathogens to microbicides [1] (developed originally from the Spaulding Classification [6]). In a U.S. Environmental Protection Agency (EPA) guidance [7], viruses have been classified into three categories, ranked from lesser to greater susceptibility to microbicides: small, non-enveloped viruses; large, non-enveloped viruses; and enveloped viruses. The generally accepted hierarchy of susceptibility of pathogens to microbicides provides to the public health communities a starting point for infection prevention and control (IPAC) measures to be used for emerging pathogens (including HFVs), per the EPA [8] and European Health authorities [9].

It is prudent, whenever possible, to confirm the efficacies of inactivation approaches predicted for emerging HFVs on the basis of the hierarchy of pathogen susceptibility by conducting empirical efficacy studies on the specific viruses. While the mechanisms of action of microbicides for viruses should apply similarly to different members of a given virus family, certain intrafamily exceptions in susceptibility are known to exist [10,11].

In the present review, we have assembled the primary literature concerning the virucidal efficacy of microbicidal active ingredients, formulated microbicidal products, hand/skin hygiene formulations, laboratory reagents, and physical inactivation approaches for inactivating HFVs. This review represents a refocusing and update of our previous review on the efficacy of microbicides against WHO Priority Disease List viruses [1] and is intended to complement recent reviews focusing primarily on inactivation methods for emerging viruses to be used for diagnostic samples or for vaccine development purposes [12,13].

## 2. Materials and Methods

The approach that we have taken for this review consisted of searching the literature (Google Scholar, PubMed) up to January 2025 for articles pertaining to virucidal efficacies of microbicidal active ingredients, microbicidal formulations, hand sanitizers, laboratory reagents, and physical inactivation approaches evaluated specifically against HFVs. Our goal was to attempt to identify and consider all the primary literature on these topics and to supplement this with the secondary literature only where the primary literature was not able to be identified.

## 3. Results

### 3.1. General Considerations

The hierarchy of pathogen susceptibility to microbicides [1,7] suggests that certain classes of microbicidal agents should display virucidal efficacy against lipid-enveloped viruses in general and therefore against the HFVs under consideration here. These include lipid-disrupting agents (alcohols, quaternary ammonium compounds such as benzalkonium chloride, phenolics such as para-chloro-meta-xylenol, detergents such as soap and nonionic surfactants, and organic acids such as citric, lactic, and salicylic acids) [14]. Protein-denaturing agents (alcohols, phenolics, oxidizers, and organic acids) inactivate enveloped viruses by degrading the spike proteins required for infection of host cells and by degrading capsid proteins [14]. Genome-degrading agents, such as alcohols and oxidizing agents, are expected to inactivate both enveloped and non-enveloped viruses [14]. Those microbicides displaying virucidal efficacy against less susceptible pathogens (such as mycobacteria, large and small non-enveloped viruses, bacterial spores/protozoan oocysts, and prions) are expected to display virucidal efficacy against HFVs as well. In the remainder of this review, we have identified the empirical testing data available to confirm the above expectations.

Physical inactivation approaches display virucidal efficacies that are less dependent upon the envelope status of the virus but rather on genome or particle size (gamma irradiation, electron beam irradiation), capsid protein make-up and spike proteins in enveloped viruses (heat), or nucleotide composition or size of the genome (ultraviolet light) [15]. The physical inactivation approaches are more typically used to render laboratory specimens safe to handle (gamma irradiation, X-ray irradiation, electron beam irradiation, heat) or for surface disinfection (ultraviolet light in the C range, UV-C) [15]. The literature addressing the empirically determined efficacies of these physical inactivation approaches for HFVs has been reviewed, and the data have been summarized along with the microbicidal inactivation data.

Virucidal activities (efficacies) of microbicidal active ingredients, formulated microbicides and hand/skin hygiene products, and physical inactivation approaches may be assessed using a variety of experimental approaches. For instance, one approach involves the examination of the efficacy for inactivating viruses intentionally deposited on model environmental surfaces (e.g., glass, plastic, or stainless-steel carriers). Such studies ideally are performed according to international standards (such as the American Society for Testing and Materials [ASTM] method ASTM E1053-20 [16]). Alternatively, the efficacy of microbicides may be tested by the addition of the active ingredients or formulations at typical use concentrations to viruses suspended in liquid matrices (suspension testing), ideally according to international standards (ASTM E1052-20 [17] or EN 14476:2013 + A2:2019 [18]). The efficacy of hand/skin hygiene agents is typically performed according to the suspension testing standards. Inactivation of viruses aerosolized into aerobiology chambers by air sanitizing sprays or devices may be evaluated according to U.S. Environmental Protection Agency guidelines and standardized protocols [19,20,21]. In each of the experimental approaches described above, the inclusion of an organic load (a mixture of organic and inorganic substances intended to mimic human pathophysiological enteric or respiratory body fluids such as mucus, saliva, or fecal matter) into the inactivation matrix is often included as a field-appropriate challenge to the inactivation approach [22]. In fact, requirements for the inclusion of such an organic load in the inactivation matrix are articulated within the international standards [16,17,18,20]. The final category of virucidal efficacy testing that has been addressed within this review pertains to inactivation approaches (chemical and physical) intended to render hemorrhagic fever virus-contaminated diagnostic samples and other laboratory specimens safe for handling within non-BSL-4 facilities. These approaches have included gamma irradiation, heat, nucleic acid extraction reagents, lysis buffers, tissue fixatives, and various types of microbicidal active ingredients and formulations.

In the sections that follow, the tables pertaining to the various families of HFVs are intended to provide only a high-level description of the inactivation efficacy results contained within the cited primary literature. That is, not all information from the cited articles has been included in the tables. Readers who are interested in a particular hemorrhagic fever virus or a particular family of HFVs and who desire additional granular detail are encouraged to obtain and review the individual articles cited. In addition, where specific formulated microbicidal or laboratory products were tested for virucidal efficacy against HFVs, the trade names of those products can be obtained by reviewing the individual articles cited.

### 3.2. Efficacy of Virucidal Approaches for Inactivating Hemorrhagic Fever Arenaviruses

Hemorrhagic fever viruses in the *Arenaviridae* family include Lassa virus (causing Lassa fever), Junin virus (Argentine hemorrhagic fever), Machupo virus (Bolivian hemorrhagic fever), Sabiá virus (Brazilian hemorrhagic fever), Lujo virus (Lujo hemorrhagic fever), and Guanarito virus (Venezuelan hemorrhagic fever). Each of these viruses is transmitted by a rodent vector [3,4]. The disease Lassa fever may be acquired through touching surfaces (fomites) contaminated with the virus, by eating contaminated food (including the rodent vector), by inhaling air contaminated with the virus (which may contain virus-contaminated urine or feces aerosolized through human activities such as sweeping) or following exposure to bodily fluids of an infected person [23]. Approaches for inactivating arenaviruses in suspension (i.e., within liquids) have been characterized quite recently, while approaches for rendering laboratory specimens safe for handling in diagnostic laboratories have been available for a longer period of time. Included in the latter category are a variety of solvents and detergents for which efficacy against a surrogate low pathogenic arenavirus (Morogor arenavirus) have been reported [24].

The results of this literature review (Table 1) indicate that knowledge gaps exist in the cases of surface hygiene and hand/skin hygiene agent efficacy for the HFVs from the arenavirus family. Until such gaps have been resolved, the efficacies of surface and hand hygiene agents shown to be effective for other enveloped viruses may be extrapolated to these arenaviral HFVs.

### 3.3. Efficacy of Virucidal Approaches for Inactivating Hemorrhagic Fever Filoviruses

Hemorrhagic fever viruses in the *Filoviridae* family [37] include the various variants of the Zaire Ebola virus (EBOV, EBOV/Mak, EBOV/May, EBOV/Kik, and EBOV/Yam-Ecr), Reston virus, Sudan virus, and Marburg virus. The filoviruses cause zoonotic infections in wild animals, and the reservoir animal is speculated to be bats [3]. During Ebola outbreaks, it has been noted that human-to-human transmission may occur through ritual handling of corpses, intrafamilial transmission, or nosocomial transmission [3]. Less is known about the transmission of the Marburg virus. A relatively great deal of research into the effectiveness of approaches for inactivating filoviruses on environmental surfaces has been reported (Table 2). Fewer data have been published on the inactivation of filoviruses in suspension (i.e., within liquids). As with the arenaviruses, multiple approaches for rendering laboratory specimens safe for handling in diagnostic laboratories have been characterized. Until the identified knowledge gaps have been resolved, the efficacy of inactivation approaches shown to be effective for other enveloped viruses in suspension testing may be extrapolated to these filoviral HFVs.

### 3.4. Efficacy of Virucidal Approaches for Inactivating Hemorrhagic Fever Flaviviruses

Hemorrhagic fever viruses in the *Flaviviridae* family include some well-known viruses that have been prevalent for some time in tropical regions of the globe, such as dengue virus and yellow fever virus, as well as more recently emerging viruses, including Kyasanur Forest disease virus (first identified in Karnataka, India in 1957), Alkhurma hemorrhagic fever virus (first isolated Jeddah, Saudia Arabia in the 1990s), and Omsk hemorrhagic fever virus (first identified around 1945 in Omsk, Russia) [57]. As with other HFVs, the global distribution of these flaviviruses is linked with the ecology of the vectors and reservoir species. In the case of yellow fever virus and dengue virus, outbreaks have occurred in areas (e.g., Brazil) where established mosquito vectors were thought to have been under control as changes in established vector ecology have occurred and the viruses have adapted to additional vector species (e.g., *Aedes albopictus*) [4]. Vector (tick) migration has also expanded the distribution of the Alkhurma hemorrhagic fever virus from its initial focus in southwestern India [4].

The results of this literature review (Table 3) indicate that some knowledge exists in the cases of surface hygiene, suspension inactivation, hand/skin hygiene agent efficacies and for approaches for rendering laboratory specimens safe for handling in diagnostic laboratories for the HFVs from the *Flaviviridae* family, although almost exclusively for yellow fever virus and dengue virus. 

### 3.5. Efficacy of Virucidal Approaches for Inactivating Hemorrhagic Fever Hantaviruses

Hemorrhagic fever viruses within the *Hantaviridae* family include the Hantaan virus, Sin Nombre virus, and Dobrava virus, for which 5–15% of cases are fatal. In contrast, Seoul, Saaremaa, and Puumala virus infections appear to be less severe, with less than 1% of cases dying from the disease. Additional named hantaviruses are listed in references [62,63]. The hantaviruses are transmitted by a variety of rodent vectors, causing in the Americas hantavirus pulmonary syndrome (HPS) and in Europe hemorrhagic fever with renal syndrome (HFRS) [64,65]. The global distributions of the various hantaviruses are determined by the host ranges of their rodent vectors.

The results of this literature review (Table 4) indicate that knowledge gaps exist in the cases of surface hygiene with microbicides and hand/skin hygiene agent efficacies for the HFVs from the *Hantaviridae* family. Until the identified knowledge gaps have been resolved, the efficacies of agents shown to be effective for other enveloped viruses in surface inactivation and hand/skin hygiene testing may be extrapolated to these hantaviral HFVs.

### 3.6. Efficacy of Virucidal Approaches for Inactivating Hemorrhagic Fever Nairoviruses

The nairovirus with the greatest public health impact on humans is the ixodid tick-borne Crimean–Congo hemorrhagic fever virus (CCHFV). CCHFV outbreaks have occurred over a geographic area including Western and Central Asia, the Middle East, Africa, and Southern Europe. The virus causes a hemorrhagic fever with a case fatality rate of 10% to 40% [71]. While it is not clear that CCHFV is transmitted during blood transfusions, investigators have proactively characterized the efficacy and fluence requirements of methylene blue and ultraviolet C or visible light inactivation systems (THERAFLEX UV-Platelets and THERAFLEX MB-Plasma, for inactivating the virus in spiked donor blood platelet concentrates or plasma, respectively) [72].

The results of this literature review (Table 5) indicate that extensive knowledge gaps exist in the case of the HFVs from the *Nairoviridae* family. The secondary literature provides some guidance (e.g., “CCHFV can be inactivated by many disinfectants including 1% hypochlorite, 70% alcohol, hydrogen peroxide, peracetic acid, iodophors, glutaraldehyde, and formalin. It can also be destroyed by UV light or pH < 6. One study found that heating at 56 °C (133 °F) for 30 min inactivated the virus, while another reported that 60 °C (140 °F) for 60 min was more effective” [73]) and similar statements have appeared in the primary literature. In each case, however, there have been no empirical details (i.e., contact times and temperatures for microbicides, strain of challenge virus, methodologies used, etc.) provided and no original references cited to substantiate the efficacy claims. A conference abstract was identified, which provided evidence that a sample inactivant (FA Lysis Buffer) led to >4 log_10_ inactivation of CCHFV when applied undiluted for 4 min contact time [74]. Efficacy data for other members of the *Nairoviridae* family, including members that appear to be relatively non-pathogenic for humans and, therefore, could be handled safely in BSL-2 or BSL-3 laboratories, are also sparse. Until the identified knowledge gaps have been resolved, the efficacies of agents shown to be effective for other enveloped viruses may be extrapolated to these nairoviral HFVs.

### 3.7. Efficacy of Virucidal Approaches for Inactivating Non-Typical Hemorrhagic Fever Paramyxoviruses

The two non-typical HFVs of the *Paramyxoviridae* family include the Nipah virus and the Hendra virus. These viruses are mentioned in the U.S. Centers for Disease Prevention and Control (CDC) website dedicated to viral hemorrhagic fevers [2], appear in the WHO Priority Disease List [5], and are specified as Priority Pathogens in the 2024 WHO Pathogens Prioritization website [77], yet have not always been included in review articles pertaining to VHF [3,4]. As the list of HFVs is fairly dynamic, and since in severe cases these paramyxoviruses can cause hemorrhage, we have considered these two non-typical HFVs within scope for this review. Both the Nipah and the Hendra viruses are transmitted via various species of *Pteropus* flying fox bats [2], and spillover animal hosts for the Nipah virus include pigs, dogs, cats, horses, and goats [78,79]. The only known spillover host for the Hendra virus is the horse [80].

While it is not clear that the Nipah virus is transmitted during blood transfusions, investigators have proactively characterized the efficacy and fluence requirements of methylene blue and ultraviolet C or visible light inactivation systems (THERAFLEX UV-Platelets and THERAFLEX MB-Plasma, for inactivating the virus in spiked donor blood platelet concentrates or plasma, respectively) [72].

The results of this literature review (Table 6) indicate that knowledge gaps exist in the cases of surface hygiene with microbicides and hand/skin hygiene agent efficacies for the HFVs from the *Paramyxoviridae* family. Until the identified knowledge gaps have been resolved, the efficacies of surface and hand/skin hygiene agents shown to be effective for other enveloped viruses may be extrapolated to the paramyxoviral HFVs.

### 3.8. Efficacy of Virucidal Approaches for Inactivating Hemorrhagic Fever Phenuiviruses

Hemorrhagic fever viruses of the *Phenuiviridae* family include Rift Valley fever virus (RVFV) in sub-Saharan Africa, the Arabian Peninsula, and certain islands of the Indian Ocean [88]; severe fever with thrombocytopenia syndrome virus (SFTSV) in East and Southeast Asia [89]; and heartland virus (HRTV) in the United States [90]. The vectors for these viruses include various mosquitos (RVFV) and ticks (SFTSV and HRTV) [88,89,90]. Methods for inactivating RVFV in mosquitos during fixation for further assay have been described [88,91].

The results of this literature review (Table 7) indicate that knowledge gaps exist in the cases of surface hygiene with microbicides and hand/skin hygiene agent efficacies for the HFVs from the *Phenuiviridae* family. In addition, insufficient coverage of the efficacy of microbicides intended for suspension inactivation of phenuiviruses is available. Until the identified knowledge gaps have been resolved, the efficacies of surface and hand/skin hygiene agents and suspension inactivation agents that were shown to be effective for other enveloped viruses may be extrapolated to the phenuiviral HFVs.

## 4. Discussion

As mentioned in the Introduction, this review of the efficacies of inactivation approaches evaluated specifically for HFVs represents an extension and refocusing of our previous review, assembled in 2021 and published in 2022 [1]. That review was focused on the efficacy of microbicidal approaches for inactivating World Health Organization (WHO) Priority Disease list viruses [5]. While the Priority Disease list in 2022 (and the more current 2024 WHO Pathogens Prioritization website [77]) included a number of diseases associated with HFVs (Crimean–Congo hemorrhagic fever, Ebola virus disease and Marburgvirus disease, Lassa fever, Nipah and henipaviral diseases, and Rift Valley fever) [5,77], these lists also included diseases caused by alphaviruses, coronaviruses, othomyxoviruses, and the flavivirus Zika virus. The latter viruses are not considered HFVs. Diseases caused by certain HFVs of the *Flaviviridae* family (yellow fever virus, Alkhumra fever virus, dengue virus) and the *Hantaviridae* family (e.g., Hantaan virus, Sin Nombre virus, and others) have not been included in the WHO Priority Disease lists. These HFVs have, with the exception of yellow fever virus, now been specified as Priority Pathogens in the 2024 WHO Pathogens Prioritization website [77].

In addition to the refocusing of this review exclusively toward HFVs, we have broadened the scope of the review to include not only the efficacies of microbicides but also the efficacies of physical inactivation approaches (such as heat, ultraviolet light in the C range, gamma irradiation, and X-irradiation), which were considered out of scope for the 2022 review [1].

We note that for these HFVs, which typically must be manipulated within BSL-3 or BSL-4 facilities, the chronologically earliest reports of efficacy have pertained to inactivation approaches used in rendering laboratory specimens safe for handling in diagnostic laboratories. This reflects the importance of diagnostic procedures in ascertaining new cases and informing epidemiological inquiries. More recently, studies focusing on inactivation approaches for surface hygiene, liquids, and hand/skin hygiene have been conducted and reported. For example, the amount of data available for these topics for arenaviruses (especially Lassa virus), filoviruses (especially Ebola virus), flaviviruses and paramyxoviruses (especially Nipah virus) has increased considerably in the relatively short time since the writing of our 2022 review [1].

A substantial knowledge base for the inactivation of filoviruses, including various variants of the Ebola virus, Reston virus, Sudan virus, and Marburg virus, is now available in the primary literature. This pertains to each of the different inactivation applications (surface hygiene, suspension inactivation, hand/skin hygiene, and decontamination of laboratory specimens). Of course, as is the case for each of the HFVs considered in this review, data on air sanitization approaches are lacking for the filoviruses. This likely reflects the relative difficulties and dangers associated with the conduct of such aerobiology studies using lethal aerosolized challenge viruses. However, efficacy studies intended to determine the in-air virucidal activity of air sanitizers against experimentally aerosolized surrogate enveloped and non-enveloped viruses have been reported [21,97]. On the basis of the hierarchy of pathogen susceptibility to microbicides, an air sanitizer demonstrating virucidal activity against airborne non-enveloped viruses should also be effective against airborne HFVs in general, as the latter are lipid-enveloped viruses [98,99].

We can rapidly summarize the most significant remaining knowledge gaps for the inactivation of HFVs based on the results of this current review. For instance, in all cases other than the filoviruses, there are limited efficacy data available pertaining to surface and hand/skin hygiene agents. The hemorrhagic fever virus family for which the least amount of inactivation efficacy information is currently available is the *Nairoviridae*. This suggests that future research should be focused on the Crimean–Congo hemorrhagic fever virus or its less pathogenic family member, the Hazara virus.

Until such data become available, it must be recognized that since each of the HFVs considered in this review are relatively large, lipid-enveloped RNA viruses, inactivation approaches that are shown to be effective for surface hygiene, liquid disinfection, or hand/skin hygiene for the filoviruses are also likely to be effective for the other families of HFVs. This position is summed up nicely by Weber et al. [100]: “However, once the nature of the emerging disease is known (i.e, bacteria, non-enveloped virus, enveloped virus), it is possible to determine the proper antiseptic and disinfectant, even in the absence of studies of the exact infectious agent. For example, an enveloped virus (e.g., Lassa, EVD, MERS-CoV, SARS-CoV, influenza A) would be inactivated by any agent active against vegetative bacteria, non-enveloped viruses, or mycobacteria” [100]. This position is also reflected in the U.S. EPA guidance Disinfectants for Emerging Viral Pathogens (EVPs): List Q [101]. The lipid envelope is an effective target for microbicides because it is a virally modified, host-cell-derived, phospholipid bilayer with associated glycoproteins, which (especially the spike glycoproteins) interact with the cellular receptors required for initiation of viral infection of the host cells [98,99].

It might be questioned by some whether hygiene (sanitization) agents are required for environmental surfaces, hand/skin, or air for HFVs known to be conveyed by insect, rodent, or mammalian vectors. While the proximate sources (reservoirs) of the various HFVs may indeed be the insect or animal vectors, there are indications that human-to-human, environmental surface-to-human, and air-to-human transmission may also be relevant for certain HFVs [102]. For example, Lassa fever may be acquired through touching surfaces (fomites) contaminated with the virus, by eating contaminated food (including the rodent vector), by inhaling air contaminated with the virus (which may contain virus-contaminated urine or feces aerosolized through human activities such as sweeping) or following exposure to bodily fluids of an infected person [23,102,103,104]. At 30% RH and 24–32 °C, the infectivity half-life of aerosolized Lassa virus Josiah strain has been reported to be 10 to 55 min [104]. The time required to reduce the surface (glass) infectious Lassa virus Josiah strain titer by 1 log_10_ has been reported to be 53 h at 30–40% RH and 20–25 °C in the dark [105]. The times required to reduce the surface infectious Lassa virus Josiah strain and Sauerwald strain titers by 1 log_10_ at room temperature were 13 h and 24 h, respectively, on high-density polyethylene surfaces and 10 h and 20 h, respectively, on stainless-steel surfaces [27]. The considerations discussed above suggest that targeted surface hygiene may represent a useful non-pharmaceutical intervention for mitigating the exposure risk to the Lassa virus.

In the case of the Ebola virus, while many cases appear to be transmitted directly through contact with the mammalian vector (fruit bats), human-to-human transmission as a result of contact with contaminated body fluids or excretions or exposure to airborne virus may also occur [102,106,107]. Indirect transmission through contact with contaminated fomites has also been considered [107,108]. The time required to reduce the surface (glass) infectious Zaire Ebola virus titer by 1 log_10_ has been reported to be 35 h at 30–40% RH and 20–25 °C in the dark [105]. Cook et al. reported similar results for EBOV/Mak [38], i.e., the time required to reduce the infectious titer by 1 log_10_ was found to be 30 h on stainless steel at 21.5 °C and 30% RH. Piercy et al. [109] reported the infectivity half-lives for aerosolized Zaire Ebola virus and Lake Victoria Marburg virus at 50–55% RH and 19–25 °C to be 104 min and 93 min, respectively. The filoviruses represent another case where the use of targeted surface hygiene in the field may help to mitigate transmission risk.

The flaviviral HFVs are transmitted exclusively through insect vectors, and studies on persistence on environmental surfaces or in the air were not identified during the literature search [102]. The hantaviral HFVs are thought to be transmitted both by direct contact with the vectors and indirectly through contact with contaminated fomites [66,102]. The nairovirus Crimean–Congo hemorrhagic fever virus primarily is transmitted directly to humans via the insect vector, although human-to-human transmission via contaminated bodily fluids is also possible, as is transmission through exposure to contaminated aerosols [73]. The persistence of infectious Hantaan virus strain 76–118 and Crimean–Congo hemorrhagic fever virus strain IbAr 10200 on aluminum discs at 20 °C was investigated by Hardestam et al. [67]. The times required to reduce the infectious titers by 1 log_10_ were found to be ~60 min for the Hantaan virus and ~90 min for the Crimean–Congo hemorrhagic fever virus [67].

Nipah and Hendra viruses are thought to be transmitted to humans through contact with the animal reservoir species or with food or fomites contaminated with excretions from the reservoir species [79,102]. Human-to-human transmission is also thought to occur in the case of the Nipah virus but has not been reported for the Hendra virus [79]. The persistence of these paramyxoviral HFVs on surfaces has not been investigated, to our knowledge. The infectivity half-life of aerosolized Nipah virus Malaysia at 19–21 °C and 42–57% RH in the dark was reported to be 47 min [82]. The phenuivirus Rift Valley fever virus is transmitted to humans via the insect vector and by contact with organs or excretions of infected animals; human-to-human transmission has not been reported [110]. Published data on the persistence of infectious RVFV on surfaces or in air were not identified during the literature search. Nipah virus is of particular concern among HFVs as it displays potential for rapid human-to-human transmission, has a high case fatality rate, and a specific antiviral treatment is lacking [111,112].

The limitations of this review include the following:

- Since the stated goal of the review was to identify inactivation efficacy information specifically for the HFVs, certain information available for family members not considered to be HFVs (especially of the *Flaviviridae* and the *Paramyxoviridae*) have not been included in the tabular summaries;
- Secondly, the emergence of new HFVs within the various virus families discussed in this review is very dynamic. Not all currently known HFVs within these families may have been mentioned. This is especially true for the arenaviruses and the hantaviruses. On the other hand, our tabular summaries provide information for two paramyxoviruses that may be considered non-typical HFVs as the resulting symptoms—though more neurological in nature—do include hemorrhages [2,113,114];
- Thirdly, the tabular information assembled represents primary empirical data only. Secondary literature information has been excluded from the tables. In addition, there are some predictive data that have not been included in the tables presented. An example of this type of data is the prediction of the susceptibilities of HFVs to ultraviolet light in the C range (UV-C, 254 nm) by Lytle and Sagripanti [115]. The predicted UV-C susceptibilities were found to be similar for the various HFVs considered (Marburg virus, Ebola virus, Hanta virus, Rift Valley fever virus, Lassa virus, and Junin virus), with decimal reduction (*D*) values (the fluence of 254 nm radiation required to inactivate 1 log_10_ of HFVs) ranging from 1.7 to 3.0 mJ/cm^2^ [115];
- Fourthly, the efficacies of certain less commonly used inactivation approaches for HFVs, including photodynamic methods (e.g., psoralen + ultraviolet light in the A range [116,117]; methylene blue + visible light [72]) and high-hydrostatic pressure inactivation [118], have not been included within the tables provided in this review as the methods are rather niche approaches for the described applications or require specialized devices that are not widely available in research and diagnostic laboratories globally.

This review represents a snapshot in time as additional inactivation efficacy studies for HFVs are being published on a continuing basis. It is hoped that, despite the various limitations discussed above, the review provides an up-to-date listing of the topical information available as of the beginning of 2025 and that readers and investigators conducting research on HFVs will find this compilation to be useful.

## Figures and Tables

**Table 1:** Efficacy of virucidal approaches for inactivating HFVs of the *Arenaviridae* family.

Virus/Strain	Active Ingredient	Inactivating Agent Type	Contact Time (min) ^a^	Concentration in Test	Efficacy (log_10_ Reduction) ^b^	Ref.
**Surface hygiene** (glass carriers)						
Lassa	N/A ^c^	UV-C (254 nm)	N/A	N/A	0.27 log_10_/mJ/cm^2^	[25]
**Suspension inactivation**						
Junin	Copper II ions, H_2_O_2_	Microbicide	Various	1 mg/L, 100 mg/L	*D* = 33 min	[26]
Lassa/Josiah	Sodium hypochlorite	Microbicide	Various	5000, 10,000 ppm	≥8 log_10_ in 5 min	[27]
Lassa/Sauerwald	Sodium hypochlorite	Microbicide	Various	5000, 10,000 ppm	≥8 log_10_ in 5 min	[27]
Lassa/Josiah	Sodium hypochlorite	Microbicide	Various	5000 ppm	≥6.7 log_10_ in 1 min	[27]
	Ethanol	Microbicide	Various	67%	≥6.7 log_10_ in 0.5 min	[28]
	Dual QAC ^d^	Microbicide	Various	2%	≥6.2 log_10_ in 0.5 min	[28]
	Accelerated H_2_O_2_	Microbicide	Various	2.5%	≥7.3 log_10_ in 1 min	[28]
Lassa	N/A	Gamma irradiation	N/A @ −60 °C	N/A	0.53 log_10_/kGy	[29]
**Hand/skin hygiene**						
Lassa/Josiah	Chloroxylenol (PCMX)	Antiseptic liquid	Various	0.12%	≥7.8 log_10_ in 1 min	[28]
**Sample disinfection procedures**						
Lassa/Josiah	Acetic acid	Sample inactivant	15	3% (pH 2.5)	≥3	[30]
Lassa	Phenol/guanidine thiocyanate	Nucleic acid extractant	10	80% of neat	≥5.0	[31]
Lujo	Phenol/guanidine thiocyanate	Nucleic acid extractant	10	80% of neat	≥5.1	[31]
Guanarito	Phenol/guanidine thiocyanate	Nucleic acid extractant	10	80% of neat	≥6.3	[31]
Machupo	Phenol/guanidine thiocyanate	Nucleic acid extractant	10	80% of neat	≥5.3	[31]
Sabiá	Phenol/guanidine thiocyanate	Nucleic acid extractant	10	80% of neat	≥6.1	[31]
Lassa/Josiah	Guanidine thiocyanate, ethanol	Lysis buffer	10, 3	50–70%, 95%	8.0	[32]
Lassa/GA391	β-propiolactone	Sample inactivant	30 @ 37 °C	0.2%	≥7	[33]
Lassa/Josiah	Formalin	Cell fixative	20 days	Neat	Complete	[34]
	N/A	Gamma irradiation	N/A @ −60 °C	N/A	~0.15 log_10_/kGy	[35]
	N/A	Heat	N/A @ 60 °C	N/A	*D* = 7.4 min	[30]
Lassa/GA391	N/A	Heat	N/A @ 60 °C	N/A	*D* = 6.0 min	[33]
Lassa/Josiah	Phenol/guanidine thiocyanate	Nucleic acid extractant	10	Neat	Complete	[36]
	Formalin	Sample inactivant	360 @ 4 °C	10%	Complete	[36]
	Phenol/guanidine thiocyanate	Nucleic acid extractant	10	75%	8	[36]

^a^ Contact times at room temperature are provided unless otherwise indicated. ^b^ Inactivation matrix was virus stock (virus in cell culture medium) unless otherwise indicated in the cited papers. ^c^ Abbreviations used: *D*, decimal reduction time (the time required to inactivate 1 log_10_ of virus); HFVs, hemorrhagic fever viruses; H_2_O_2_, hydrogen peroxide; kGy, kiloGrays; min, minutes; mJ/cm^2^, milliJoules per square centimeter; N/A, not applicable; PCMX, para-chloro-meta-xylenol; ppm, parts per million; QAC, quaternary ammonium compound; UV-C, ultraviolet light in the C range. ^d^ Ingredients shown in blue font are incorporated into a formulated product. The cited papers may be consulted to obtain the product trade names.

**Table 2:** Efficacy of virucidal approaches for inactivating HFVs of the *Filoviridae* family.

Virus/Strain	Active Ingredient	Inactivating Agent Type	Contact Time (min) ^a^	Concentration in Test	Efficacy (log_10_ Reduction) ^b^	Ref.
**Surface hygiene** (steel or aluminum carriers)						
EBOV/Mak	Sodium hypochlorite	Microbicide	5	0.5%	≥6.6	[38]
	Sodium hypochlorite	Microbicide	5	0.5%	≥6.8	[39]
	Sodium hypochlorite	Microbicide	15	0.5%	≥2.0	[40]
	Sodium hypochlorite	Microbicide	15	0.5%	<1	[40]
	Sodium hypochlorite	Microbicide	5	0.5%	≥5.1	[41]
	Sodium hypochlorite ^d^	Pre-impregnated wipe	0.08	1%	6.3	[42]
	Ethanol	Alcohol	5	67%	≥7.3	[38]
	Ethanol	Alcohol	2.5	70%	≥6.8	[39]
	Ethanol	Alcohol	5	70%	≥6.9	[41]
	Ethanol	Disinfectant spray	5	58%	≥4.5	[41]
	Ethanol	Pre-impregnated wipe	0.08	66.5%	6.6	[42]
	Ethanol	Alcohol	2	70%	1.7	[40]
	Ethanol	Alcohol	2	70%	<1	[40]
	Peracetic acid	Microbicide		5%	≥1.0	[40]
	Peracetic acid	Microbicide		5%	≥2.0	[40]
	Chloroxylenol (PCMX) ^c^	Microbicide	5	0.48%	≥5.1	[41]
	H_2_O_2_	Pre-impregnated wipe	1	2.5%	6.4	[42]
	H_2_O_2_, peroxyacetic acid	Microbicide	5	Net	2.6	[40]
	H_2_O_2_, peroxyacetic acid	Microbicide	5	Neat	<1	[40]
	QAC	Microbicide	10	1.5%	<1	[40]
	QAC	Pre-impregnated wipe	1	As supplied	6.6	[42]
	QAC	Pre-impregnated wipe	0.08	5%	6.0	[42]
EBOV/May	Sodium hypochlorite	Microbicide	5	0.5%	≥6.6	[39]
	Ethanol	Alcohol	1	70%	≥6.6	[39]
EBOV/Kik	Sodium hypochlorite	Microbicide	5	0.5%	≥6.5	[39]
	Ethanol	Alcohol	1	70%	≥6.5	[39]
EBOV/Yam-Ecr	Sodium hypochlorite	Microbicide	10	0.75%	≥6.5	[43]
	Alcohol formulation	Microbicide	10	50%	5.3	[43]
	QAC, alcohol	Microbicide	10	1.5%	2.5	[43]
	QAC, alkylamine	Microbicide	10	2.5%	4.2	[43]
**Suspension inactivation**						
EBOV/Mak	Sodium hypochlorite	Microbicide	0.33	5 ppm	≥4.2	[44]
EBOV	Povidone-iodine	Microbicide	0.25	1:10	≥5.5	[45]
	N/A	Gamma irradiation	N/A @ −60 °C	N/A	0.84 log_10_/kGy	[29]
	N/A	Gamma irradiation	N/A	N/A	0.44 log_10_/kGy	[46]
Sudan	N/A	Gamma irradiation	N/A	N/A	0.44 log_10_/kGy	[46]
Marburg	N/A	Gamma irradiation	N/A @ −60 °C	N/A	0.68 log_10_/kGy	[29]
**Hand/skin hygiene**						
EBOV/Mak	Chloroxylenol (PCMX)	Antiseptic liquid	1	0.48%	≥4.8	[47]
	Salicylic acid, citric acid	Liquid hand wash	0.5	1:4	4.8	[48]
EBOV	Povidone-iodine	Skin cleanser	0.5	1:10	≥4.5	[45]
	Povidone-iodine	Surgical scrub	0.25	1:10	≥5.5	[45]
	Povidone-iodine, alcohol	Skin cleanser	0.25	Neat	≥5.7	[45]
EBOV/May	Ethanol, H_2_O_2_	WHO formulation I hand rub (original)	0.5	32%, 0.05%	≥5	[49]
	2-Propanol, H_2_O_2_	WHO formulation II hand rub (original)	0.5	24%, 0.04%	≥5	[49]
**Sample disinfection procedures**						
EBOV	nonionic surfactants	Detergent	60	0.1%	4	[50]
EBOV/Mak	nonionic surfactants	Detergent	60	0.1%	≥3	[51]
	Phenol/guanidine thiocyanate	Nucleic acid extractant	10	80% of neat	≥5.7	[31]
	Sodium dodecyl sulfate	Detergent	60	0.1%	≥3	[51]
	Sodium dodecyl sulfate	Detergent	60	0.1%	~1	[51]
	Guanidine thiocyanate, ethanol	Lysis buffer	10, 3	50–70%, 95%	7.4 to 7.6	[32]
Reston	Phenol/guanidine thiocyanate	Nucleic acid extractant	10	80% of neat	≥5.1	[31]
Sudan	Phenol/guanidine thiocyanate	Nucleic acid extractant	10	80% of neat	≥5.1	[31]
EBOV/May	Acetic acid	Sample inactivant	15	3% (pH 2.5)	≥3	[30]
	N/A	Heat	N/A @ 60 °C	N/A	*D* = 4.4 min	[30]
	N/A	Heat	10 @ >99 °C	N/A	Complete	[36]
	N/A	Heat	10 @ >99 °C	N/A	>8	[36]
	Phenol/guanidine thiocyanate	Nucleic acid extractant	10	N/A	Complete	[36]
	Phenol/guanidine thiocyanate	Nucleic acid extractant	10	N/A	8	[36]
	N/A	Gamma irradiation	N/A @ −60 °C	N/A	~0.3 log_10_/kGy	[35]
	N/A	Gamma irradiation	N/A @ −60 °C	N/A	0.31 log_10_/kGy	[52]
EBOV	Formalin	Sample inactivant	360 @ 4 °C	10%	8.4	[36]
	nonionic surfactants	Sample inactivant	20	0.8%	Incomplete	[53]
	nonionic surfactants + phenol/guanidine thiocyanate	Nucleic acid extractant	10	0.08%	Complete (6.0)	[53]
EBOV	Phenol/guanidine thiocyanate	Nucleic acid extractant	10	80% of neat	≥5.3	[54]
	Phenol/guanidine thiocyanate	Nucleic acid extractant	10	80% of neat	≥5.3	[55]
	Neutral buffered formalin	Tissue fixative	≤1 week	10%	9.08	[55]
	Osmium tetroxide	Tissue fixative	60	1%	4.9	[55]
EBOV/Kik	Phenol/guanidine thiocyanate	Nucleic acid extractant	10	80% of neat	Incomplete (<6)	[56]
	Phenol/guanidine thiocyanate + ethanol	Nucleic acid extractant	10	80% of neat	6	[56]
Marburg/Ci67	Phenol/guanidine thiocyanate	Nucleic acid extractant	10	80% of neat	≥6.5	[31]
	Phenol/guanidine thiocyanate	Nucleic acid extractant	10	80% of neat	≥6.8	[54]
	N/A	Gamma irradiation	N/A @ −60 °C	N/A	~0.3 log_10_/kGy	[35]
Marburg/371Bat	Guanidine thiocyanate, ethanol	Lysis buffer	10, 3	50–70%, 95%	6.5	[32]
Marburg/Musokee	Acetic acid	Sample inactivant	15	3% (pH 2.5)	≥3	[30]
	Phenol/guanidine thiocyanate	Nucleic acid extractant	10	80% of neat	≥6.7	[54]
	N/A	Heat	N/A @ 60 °C	N/A	*D* = 7.4 min	[30]

^a^ Contact times at room temperature are provided unless otherwise indicated. ^b^ Inactivation matrix was virus stock (virus in cell culture medium) unless otherwise indicated in the cited papers. ^c^ Abbreviations used: *D*, decimal reduction time (the time required to inactivate 1 log_10_ of virus); HFVs, hemorrhagic fever viruses; H_2_O_2_, hydrogen peroxide; kGy, kiloGrays; min, minutes; N/A, not applicable; PCMX, para-chloro-meta-xylenol; ppm, parts per million; QAC, quaternary ammonium compound. ^d^ Ingredients shown in blue font are incorporated into a formulated product. The cited papers may be consulted to obtain the product trade names.

**Table 3:** Efficacy of virucidal approaches for inactivating HFVs of the *Flaviviridae* family.

Virus/Strain	Active Ingredient	Inactivating Agent Type	Contact Time (min) ^a^	Concentration in Test	Efficacy (log_10_ Reduction) ^b^	Ref.
**Surface hygiene** (stainless steel carriers)						
Yellow fever/17D	1-Propanol, 2-propanol, ethanol ^d^	Microbicide	0.5	80%	≥3.4	[58]
	Ethanol, 2-propanol	Microbicide	0.5	80%	≥3.4	[58]
	Glutaraldehyde, QAC ^c^	Microbicide	5	0.5%	≥2.4	[58]
	Glutaraldehyde, QAC	Microbicide	5	0.5%	≥2.4	[58]
	H_2_O_2_	Microbicide	0.5	80%	1.7	[58]
**Suspension inactivation**						
Yellow fever/17D	Ethanol	Microbicide	0.5	40%	≥5.5	[58]
	2-Propanol	Microbicide	0.5	20%	≥5.5	[58]
	H_2_O_2_	Microbicide	120	3%	≥6	[59]
Yellow fever	N/A	Heat	N/A @ 60 °C	N/A	*D* = 13.2 min	[60]
Alkhumra fever/AHFV/997/NJ/09/SA	N/A	Heat	N/A @ 60 °C	N/A	*D* = 0.32 min	[61]
	N/A	Heat	N/A @ 45 °C	N/A	*D* = 11.1 min	[61]
**Hand/skin hygiene**						
Yellow fever/17D	Ethanol, H_2_O_2_	WHO formulation I hand rub (original)	0.5	40%	≥5.5	[58]
	2-Propanol, H_2_O_2_	WHO formulation II hand rub (original)	0.5	30%	≥5.5	[58]
**Sample disinfection procedures**						
Dengue serotype 1/Hawaii	Phenol/guanidine thiocyanate	Nucleic acid extractant	10	80% of neat	≥4.1	[54]
Dengue serotype 2/S16803	Phenol/guanidine thiocyanate	Nucleic acid extractant	10	80% of neat	≥5.0	[54]
Dengue serotype 3/CH53489	Phenol/guanidine thiocyanate	Nucleic acid extractant	10	80% of neat	≥3.3	[54]
Dengue serotype 4/H241	Phenol/guanidine thiocyanate	Nucleic acid extractant	10	80% of neat	≥1.9	[54]
Dengue serotype 2/92T	Phenol/guanidine thiocyanate	Nucleic acid extractant	10	80% of neat	≥5.1	[31]
	Acetone	Cell fixative	1440 @ 4°C	50%	Incomplete	[62]
	Paraformaldehyde	Cell fixative	180	4%	Complete	[62]
Dengue/serotypes 1–4	N/A	Heat	30 @ 56 °C	N/A	≥3.4 log_10_	[63]

^a^ Contact times at room temperature are provided unless otherwise indicated. ^b^ Inactivation matrix was virus stock (virus in cell culture medium) unless otherwise indicated in the cited papers. ^c^ Abbreviations used: *D*, decimal reduction time (the time required to inactivate 1 log_10_ of virus); HFVs, hemorrhagic fever viruses; H_2_O_2_, hydrogen peroxide; min, minutes; N/A, not applicable. ^d^ Ingredients shown in blue font are incorporated into a formulated product. The cited papers may be consulted to obtain the product trade names.

**Table 4:** Efficacy of virucidal approaches for inactivating HFVs of the *Hantaviridae* family.

Virus/Strain	Active Ingredient	Inactivating Agent Type	Contact Time (min) ^a^	Concentration in Test	Efficacy (log_10_ Reduction) ^b^	Ref.
**Surface hygiene** (glass carriers)						
Puumala	N/A ^c^	Heat	60 @ 56 °C	N/A	Incomplete (<5.8)	[66]
Tula	N/A	Heat	60 @ 56 °C	N/A	Complete (5.9)	[66]
**Suspension inactivation**						
Hantaan/76–118	Ethanol	Microbicide	2	30% to 70%	Complete (6.5)	[67]
Puumala	N/A	Heat	15 @ 56 °C	N/A	Complete (5.8)	[66]
Puumala/CG1829	Ethanol	Microbicide	30	70%	≥3.7	[68]
	Peracetic acid	Microbicide	10	1%	≥3.7	[68]
	Sodium hypochlorite	Microbicide	10	1% (1000 ppm)	≥3.7	[68]
	Chlorine dioxide ^d^	Microbicide	10	1%	≥3.7	[68]
	Chloroxylenol (PCMX)	Microbicide	10	1%	≥3.7	[68]
	Sodium p-toluenesulfonchloramide, trihydrate	Microbicide	10	1%	≥3.7	[68]
	Potassium peroxymonosulfate	Microbicide	10	1%	≥3.7	[68]
Tula	N/A	Heat	15 @ 56 °C	N/A	Complete (5.9)	[66]
**Hand/skin hygiene**						
No primary literature was identified						
**Sample disinfection procedures**						
Hantaan/76–118	Methanol	Sample fixative	8	Absolute	≥5.5	[69]
	Paraformaldehyde	Sample fixative	20	1%	≥4.3	[69]
	Acetone/methanol	Sample fixative	10	50%/50%	≥5.6	[69]
	Lysis buffer + detergent	Sample extractant	10	1% detergent	≥2	[69]
	N/A	UV-C (254 nm)	N/A	500 mJ/cm^2^	Incomplete (3.9)	[69]
	N/A	UV-C (254 nm)	N/A	1400 mJ/cm^2^	Complete (≥5.2)	[69]
Puumala/CG1829	ß-propiolactone	Vaccine inactivant	30	0.025%	≥5.4	[70]
	Formaldehyde	Vaccine inactivant	14	0.025%	≥5.4	[70]

^a^ Contact times at room temperature are provided unless otherwise indicated. ^b^ Inactivation matrix was virus stock (virus in cell culture medium) unless otherwise indicated in the cited papers. ^c^ Abbreviations used: HFVs, hemorrhagic fever viruses; mJ/cm^2^, milliJoules per square centimeter; min, minutes; N/A, not applicable; PCMX, para-chloro-meta-xylenol; ppm, parts per million; UV-C, ultraviolet light in the C range. ^d^ Ingredients shown in blue font are incorporated into a formulated product. The cited papers may be consulted to obtain the product trade names.

**Table 5:** Efficacy of virucidal approaches for inactivating HFVs of the *Nairoviridae* family.

Virus/Strain	Active Ingredient	Product Type	Contact Time (min) ^a^	Concentration in Test	Efficacy (log_10_) ^b^	Ref.
**Surface hygiene**						
No primary literature was identified						
**Hand/skin hygiene**						
No primary literature was identified						
**Suspension inactivation**						
CCHFV/IbAr 10200	Ethanol	Microbicide	2	20% to 70%	Complete (4.6)	[67]
**Sample disinfection procedures**						
CCHFV/IbAr 10200	N/A ^c^	Heat	60 @ 60 °C	N/A	3.5	[75]
Hazara/JC280 ^d^	N/A	X-irradiation	N/A	N/A	1.04 log_10_/kGy	[76]

^a^ Contact times at room temperature are provided unless otherwise indicated. ^b^ Inactivation matrix was virus stock (virus in cell culture medium) unless otherwise indicated in the cited papers. ^c^ Abbreviations used: CCHFV, Crimean–Congo hemorrhagic fever virus; HFVs, hemorrhagic fever viruses; kGy, kiloGrays; min, minutes; N/A, not applicable. ^d^ Hazara virus is a nairovirus that has not been found to be pathogenic for humans and has been used as a CCHFV surrogate in inactivation efficacy testing [74].

**Table 6:** Efficacy of virucidal approaches for inactivating non-typical HFVs of the *Paramyxoviridae* family.

Virus/Strain	Active Ingredient	Product Type	Contact Time (min) ^a^	Concentration in Test	Efficacy (log_10_) ^b^	Ref.
**Surface hygiene**						
No primary literature was identified						
**Suspension inactivation**						
Nipah/Bangladesh	Dual QAC ^c,d^	Microbicide	0.5	0.28%	>4	[81]
	Dual QAC	Microbicide	1	0.095%	>4	[81]
	Ethanol	Microbicide	0.25	19%	>4	[81]
Nipah/Malaysia	Sodium hypochlorite	Microbicide	1	10%	≥5.5	[82]
	Ethanol	Microbicide	1	80%	≥5.5	[82]
Nipah	Low pH	HCl	60	pH 2	≥3	[83]
	High pH	NaOH	60	pH 12	≥3	[83]
Hendra	Low pH	HCl	60	pH 3	≥3	[83]
	High pH	NaOH	60	pH 12	≥3	[83]
**Hand/skin hygiene agents**						
No primary literature was identified						
**Sample disinfection procedures**						
Nipah/Malaysia	Formalin	Fixative	1440	10%	≥5	[82]
	Paraformaldehyde	Sample inactivant	30	4%	Complete	[84]
	N/A	Gamma irradiation	N/A @ −60 °C	N/A	~0.15 log_10_/kGy	[35]
Nipah/Ma-JMR-01-98	N/A	Heat	60 @ 56 °C	N/A	≥4.9	[85]
	N/A	Heat	30 @ 60 °C	N/A	≥4.9	[85]
	N/A	UV-C (312 nm)	30	N/A	0.0075 log_10_/mJ/cm^2^	[85]
	N/A	UV-C (312 nm)	30	N/A	0.0033 log_10_/mJ/cm^2^	[85]
Nipah	Paraformaldehyde	Sample inactivant	15	4%	Complete	[86]
	Formalin	Tissue fixative	2880	10%	Complete	[86]
Nipah/Bangladesh	Phenol/guanidine thiocyanate	Nucleic acid extractant	10	Neat	Complete	[36]
	Formalin	Sample inactivant	360 @ 4 °C	10%	Complete	[36]
	Phenol/guanidine thiocyanate	Nucleic acid extractant	10	75%	7.1	[36]
	N/A	Heat	30 @ 60 °C	N/A	Complete	[87]
Hendra	N/A	Gamma irradiation	N/A @ −60 °C	N/A	~0.15 log_10_/kGy	[35]

^a^ Contact times at room temperature are provided unless otherwise indicated. ^b^ Inactivation matrix was virus stock (virus in cell culture medium) unless otherwise indicated in the cited papers. ^c^ Abbreviations used: HFVs, hemorrhagic fever viruses; kGy, kiloGrays; min, minutes; mJ/cm^2^, milliJoules per square centimeter; N/A, not applicable; QAC, quaternary ammonium compound; UV-C, ultraviolet light in the C range. ^d^ Ingredients shown in blue font are incorporated into a formulated product. The cited papers may be consulted to obtain the product trade names.

**Table 7:** Efficacy of virucidal approaches for inactivating HFVs of the *Phenuiviridae* family.

Virus/Strain	Active Ingredient	Product Type	Contact Time (min) ^a^	Concentration in Test	Efficacy (log_10_) ^b^	Ref.
**Surface hygiene**						
No primary literature was identified						
**Hand/skin hygiene**						
No primary literature was identified						
**Suspension inactivation**						
RVFV	N/A ^c^	Heat	N/A @ 70 °C	N/A	*D* = 2.2 min	[92]
	N/A	Heat	N/A @ 80 °C	N/A	*D* = 1.2 min	[92]
RVFV/Menya/Sheep/258	N/A	Heat	N/A @ 95 °C	N/A	*D* = 0.34 min	[92]
	β-propiolactone	Vaccine inactivant	240	3.5 mM	≥5.5	[93]
	Formalin ^d^	Vaccine inactivant	360	0.2%	≥5.4	[93]
**Sample disinfection procedures ^e^**						
RVFV/AH-501	Phenol/guanidine thiocyanate	Nucleic acid extractant	10	80% of neat	≥6.8	[31]
RVFV	Formaldehyde	Cell fixative	1080	0.4%	≥7.1	[94]
RVFV/MP12	Formalin	Cell fixative	210 @ 4 °C	Neat	Complete	[34]
RVFV/ArB 1976	N/A	Heat	60 @ 60 °C	N/A	6.7	[75]
RVFV	N/A	Heat	5 @ 95 °C	N/A	Complete	[92]
RVFV/MP12	Guanidine isothiocyanate	Nucleic acid extractant	Not given	Neat	Complete	[95]
RVFV/ZH501	Guanidine isothiocyanate	Nucleic acid extractant	Not given	Neat	Complete	[95]
RVFV/MP12	Formaldehyde	Cell fixative	20 @ 4 °C	4.2%	Complete	[95]
RVFV/ZH501	Formaldehyde	Cell Fixative	20 @ 4 °C	4.2%	Complete	[95]
	N/A	Gamma irradiation	N/A @ −60 °C	N/A	~0.15 log_10_/kGy	[35]
	N/A	X-irradiation	N/A	N/A	0.38 log_10_/kGy	[76]
	N/A	X-irradiation	N/A @ −30 °C	N/A	0.26 log_10_/kGy	[76]
SFTSV/YGI	N/A	Heat	N/A0 @ 60 °C	N/A	≥4.8	[89]
	N/A	UV-C (312 nm)	30 @ 60 °C	52 mJ/cm^2^	≥4.8	[89]
	N/A	UV-C (312 nm)	30 @ 60 °C	1548 mJ/cm^2^	≥4.5	[89]

^a^ Contact times at room temperature are provided unless otherwise indicated. ^b^ Inactivation matrix was virus stock (virus in cell culture medium) unless otherwise indicated in the cited papers. ^c^ Abbreviations used: *D*, decimal reduction time (the time required to inactivate 1 log_10_ of virus); HFVs, hemorrhagic fever viruses; kGy, kiloGrays; mJ/cm^2^, milliJoules per square centimeter; min, minutes; N/A, not applicable; RVFV, Rift Valley fever virus; SFTSV, severe fever with thrombocytopenia syndrome virus; UV-C, ultraviolet light in the C range. ^d^ Ingredients shown in blue font are incorporated into a formulated product. The cited papers may be consulted to obtain the product trade names. ^e^ A set of validated procedures for inactivation of Rift Valley fever virus has also been proposed by Confort et al. [96].

## Data Availability

This is a review article. All original data may be obtained from the literature cited.
